# The interplay between cognition, depression, anxiety, and sleep in primary Sjogren’s syndrome patients

**DOI:** 10.1038/s41598-022-17354-1

**Published:** 2022-08-01

**Authors:** Radjiv Goulabchand, Elodie Castille, Sophie Navucet, Damien Etchecopar-Etchart, Aurélie Matos, Alexandre Maria, Laure Anne Gutierrez, Alain Le Quellec, Nicolas Menjot de Champfleur, Audrey Gabelle, Philippe Guilpain

**Affiliations:** 1 Internal Medicine Department, CHU Nîmes, Place du Pr Debre, 30029 Nîmes Cedex, France; 2 University of Montpellier, Montpellier, France; 3 Inserm U1183, Institute for Regenerative Medicine and Biotherapy, St Eloi Hospital, Montpellier, France; 4 Department of Internal Medicine and Multi-Organic Diseases, St Eloi Hospital, Montpellier University Hospital, Montpellier, France; 5 Department of Neurology, Memory Research and Resources Alzheimer Center, Gui de Chauliac Hospital, Montpellier University Hospital, Montpellier, France; 6 INM, Inserm, University of Montpellier, Montpellier, France; 7 Faculté de Médecine-Secteur Timone, EA 3279: CEReSS-Centre d’Etude et de Recherche sur les Services de Santé et la Qualité de vie, Aix-Marseille Université, Marseille, France; 8 Fondation FonaMental, Créteil, France; 9 Department of Neuroradiology, I2FH, Institut d’Imagerie Fonctionnelle Humaine, Gui de Chauliac Hospital, Montpellier University Hospital, Montpellier, France; 10 Laboratoire Charles Coulomb, University of Montpellier, Montpellier, France

**Keywords:** Autoimmune diseases, Dementia

## Abstract

Primary Sjögren’s syndrome (pSS) is an autoimmune disease with frequent neurological involvement. Memory complaints are common, but their precise patterns remain unclear. We wanted to characterize patterns of neurocognitive profiles in pSS patients with cognitive complaints. Only pSS patients with memory complaints were included, prospectively. Cognitive profiles were compiled through a comprehensive cognitive evaluation by neuropsychologists. Evaluations of anxiety, depression, fatigue, sleep disorders and quality of life were performed for testing their interactions with cognitive profiles. All 32 pSS patients showed at least borderline cognitive impairment, and 17 (53%) exhibited a pathological cognitive profile: a hippocampal profile (37%), a dysexecutive profile (22%), and an instrumental profile (16%) (possible overlap). Regarding the secondary objectives: 37% of patients were depressed, and 48% exhibited a mild-to-severe anxiety trait. Sleep disorders were frequent (excessive daytime sleepiness (55%), high risk for sleep apnea (45%), and insomnia (77%)). Cognitive impairments could not be explained alone by anxiety, depression or sleep disorders. Fatigue level was strongly associated with sleep disorders. Our study highlights that cognitive complaints in pSS patients are supported by measurable cognitive impairments, apart from frequently associated disorders such as depression, anxiety or sleep troubles. Sleep disorders should be screened.

## Introduction

Primary Sjögren’s syndrome (pSS) is an autoimmune disease with glandular (sicca syndrome) and extra-glandular B lymphocytic infiltration, not associated with concomitant autoimmune diseases such as rheumatoid arthritis or systemic lupus erythematosus. Among extra-glandular manifestations of pSS, psycho-cognitive troubles are frequently observed^1,2^, with a prevalence varying from 10 to 60%^3–5^. These cognitive symptoms have a negative impact on patient’s quality of life^6,7^, may contribute to fatigue (a major burden in pSS), or underlie depression^8^, and thus impact the burden on the health system^1^. Most patients report “brain fog” symptoms^2^, described as memory lapses, forgetfulness, mental confusion, diminished ability to concentrate, to organize, or to anticipate future events. The precise neuropsychological pattern of pSS is not fully deciphered, however, executive functions are often abnormal^9,10^. The interplay between cognitive complaints, cognitive impairment, fatigue, depression^11^, sleep disorders^12^, and pain is not fully understood in pSS.

Thus, we aimed to describe the precise patterns of neurocognitive profiles from a cohort of pSS patients with cognitive complaints via an extensive and comprehensive neuropsychological approach. In addition, we deciphered the relationships between cognitive profile, fatigue, depression, anxiety, sleep disorders and self-reported quality of life, and herein propose a tailored management of cognitive complaints in pSS patients.

## Materials and methods

### Patients

We prospectively included all consecutive adult pSS patients with unexplained cognitive complaints, characterized by memory loss, attention troubles, or “brain fog”, followed between November 2016 and January 2019 in the department of internal medicine of Montpellier university hospital. Patients with pre-existing conditions or treatments interfering with cognitive complaints were excluded (i.e. diagnosed depression, or damage of central nervous system). The diagnosis of pSS was assessed by experienced physicians, based on ACR/EULAR (American College of Rheumatology/EUropean League Against Rheumatism) diagnosis criteria^13^. The history of the disease, including clinical, biological (presence of antinuclear antibodies (ANA), and anti-SSa, and/or anti-SSb), pathological, and radiological findings, such as treatment strategies and main cardiovascular risk factors were recorded. Systemic involvement of the disease was defined by lung, cardiac, kidney, hematological or neurological involvements of the disease. The level of activity of pSS was assessed according to the EULAR Sjögren’s Syndrome Disease Activity Index (ESSDAI)^14^, and the patient-reported evaluation used the EULAR Sjögren’s Syndrome Patient Reported Index (ESSPRI), including a self-evaluation of pain, fatigue, and sicca syndrome^14^. Both were done at the moment of the baseline cognitive assessments.

### Cognitive assessments

A large battery of cognitive tests was performed including: educational level, ranging from 1 to 7^15^; the Mac Nair scale; the French cognitive complaint questionnaires (QPC)^16^; Mini-mental state (MMSE)^17^; the French version of the Free and Cued Reminding Selective Test (FCRST)^18^; digit span; Digit Symbol Substitution Test (DSST) (subpart of Wechsler Adult Intelligence Scale-Fourth Edition, WAIS-IV)^19^; Trail making test (TMT)^20^; Rey-Osterrieth complex figure test^21^; Verbal Fluency Test (VFT); Categorical Naming Test (CNT)^22^; the Stroop Color and Word and Interference Test^23^; Visual Object and Space Perception (VOSP)^24^; the picture naming test (D080)^25^; and the praxis’ tests^26^. A list of all tests is available in Supplementary Table 1.

The result of each test was classified into three categories: (1) normal; (2) borderline scores, without clinical impact; (3) and pathological scores. As no pathological cognitive threshold was validated in pSS population, we determined the threshold using previous validated values in < 60yo-adults, using Z-scores for the neuropsychological tests done or percentiles. Details on pathological and borderline thresholds for each test and survey are available in Supplementary Table 2. These thresholds were established including the age, sex and educational level of each patient. Some cognitive functions abnormalities may overlap on several tests (Supplementary Tables 1 and 2).

Based on this extensive evaluation, and according to the expertise of the neuropsychologists’ team, pSS patients’ cognitive skills were classified into three categories: (1) global memory impairment (at least one confirmed deficit in FCRST or digit span); (2) global executive functions impairment (a defect in at least three executive components among mental flexibility, planning, inhibition, initiation and/or spontaneous recovery, assessed through DSST, TMT, Rey-Osterrieth complex figure test, VFT, CNT and Stroop test); or (3) global instrumental functions impairment (impairment in one of the different functions among gnosis, praxis and/or language, assessed through praxis’ test, VOSP and DO80). A global neurocognitive profile was established integrating those results. The global cognitive profile was classified as pathological if ≥ 1 subcategory (memory, executive or instrumental functions) was classified as pathological; and the global cognitive profile was classified as borderline if ≥ 1 subcategory was classified as borderline.

### Fatigue and sleep disorders

Because of the broad spectrum of fatigue manifestations, fatigue was evaluated with three dimensions: (1) the Chalder Fatigue scale (14 self-reported questions assessing physical and mental fatigue, a score ≥ 9 assessing pathological fatigue for the youngers)^27^; (2) the Multidimensional Fatigue Inventory (MFI) including 20 items about general fatigue, physical fatigue, mental fatigue, reduction of daily activities, and decreased motivation^28^; (3) the ESSPRI, including a Likert scale for fatigue, from 0 to 10^14^. Results were adjusted to 100 for ease of plotting on radar charts. In addition, pain level was evaluated through the ESSPRI subscale^14^. Sleep disorders were described by the Berlin questionnaire (probability of sleep apnea disorder)^29^; the Epworth Sleepiness Scale (ESS) assessing the global quality of the sleep according to daily sleepiness^30^; and Insomnia Severity Index (ISI) used for the severity of insomnia^31^.

### Psychiatric comorbidities

Depression was evaluated by the French version of the 21-item self-questionnaire Beck Depression Inventory version II (BDI)^32^: depression is diagnosed when score > 13 and a severe depression when > 29. Anhedonia was evaluated through Chapman’s scale^33^. We evaluated self-esteem through the Rosenberg self-esteem scale^34^: low or very low self-esteem < 31/41 points. Anxiety was assessed by the French version of State Trait Anxiety Inventory (STAI)^35^, which is representative of the “usual” anxiety state of the subject.

### Quality of life

To analyze the health-related quality of life (HR-QoL), we used the EuroQol 5 Dimensions (EQ-5D) and the Short-Form 36 (SF-36), widely used in autoimmune diseases. The EQ-5D refers to five dimensions of HR-QoL: mobility; independence; daily activities; pain or discomfort; anxiety or depression feelings (poor level of HR-QoL was defined with a Time Trade-Off value < 0.5). The self-reported SF-36 assesses patients’ quality of life (QoL) in eight domains: physical functioning, social functioning (social limitations), role limitations due to physical health, bodily pain, general health, mental health, role limitations due to emotional problems, health thinking (general health perception). The score is summarized into two levels: the physical component score (PCS), and the mental component score (MCS). Lower scores represent worse QoL.

### MRI investigations

Brain MRIs were performed concomitantly to cognitive tests, in a subgroup of volunteer pSS patients (all patients were offered a brain MRI) (3 T [MAGNETOM Skyra, Siemens Healthcare, Erlangen, Germany], T1- and T2-weighted imaging, and the FLAIR sequence). Brain atrophy and white matter hyperintensity lesions load were evaluated by visual rating. Fazekas’ scale quantifies the white matter hyperintensities attributed to the chronic small vessel ischemia (vascular burden). A score > 0 is considered as pathological. Scheltens’ score aims at estimating the hippocampal atrophy: a score of 1 is considered as pathological (beginning atrophy).

### Statistical analysis

All quantitative data were expressed as mean ± [standard deviation], and qualitative variable were presented as proportion (%). Between-group comparisons of quantitative variables were performed using the Student T-test if both distributions followed a normal distribution and the Mann–Whitney U-test if not. The Chi2 test (or Fisher test when appropriate) was used to compare categorical variables frequencies in the two group. *p* values under 0.05 were considered as significant. Correlation analysis were performed with Pearson correlation coefficient because of normality of the 2 distributions compared. We compared demographical and pSS characteristics, the prevalence of fatigue, pain, psychiatric disorders, and sleep disorders, and brain MRI results, between pSS patients with and without pathological cognitive profile, patients with and without poor self-reported quality of life (according to EQ-5D), and patients with or without pathological fatigue score (according to Chalder Fatigue scale). Missing data were not included in the analysis. Data and statistical analyses were performed using SAS software (SAS® 9.4; SAS institute inc., Cary, NC, USA).

### Ethical concern

Our study was conducted in accordance with the local ethical committee of the University Hospital center of Montpellier (Accreditation number 198711; Institutional review board 2018_IRB-MTP_06-08), and in accordance with the Declaration of Helsinki. All included subjects gave informed written consent to participate in the study.

## Results

### Demographical, immunological, and radiological characteristics of pSS patients

All consecutive pSS volunteers with unexplained cognitive complaints were included: 32 (31 female and 1 male) (Supplementary Fig. 1). None of them withdrew participation. Their main characteristics are listed in Table 1. The median age was 58 years old [51–69], with a mean disease duration of 11.8 years [± 8.3]. Fifty-nine percent of the population had an educational level of bachelor or higher [2–7]. Seventy-five percent of patients were positive for ANA (37.5% for anti-SSa and/or SSb). Seven patients had lung involvement of the disease (21.9%). Five patients (15.6%) had peripheral neurological symptoms (4 cases of small fiber neuropathy and 1 case of ganglionopathy). Brain MRIs analysis revealed Fazekas’ score > 0 in 20/25 patients with available data (80%). Scheltens’ scale was at 1 in 6/24 patients (25%). Patients with brain MRIs did not differ from those without brain MRIs on pSS characteristics and neuropsychological assessments.

**Table 1:** Demographical, clinical, immunological characteristics of the population of primary Sjögren's syndrome patients with cognitive complaints.

			Available data
**Demographical characteristics**			
Female (n, %)	31	97.0%	32
Age at diagnosis, years (median, [IQR])	54	[45–63]	32
Age at neurocognitive evaluation, years (median, [IQR])	58	[51–69]	32
Educational level (n, %)			
Illiterate	0	0.0%	32
Primary education level	7	21.9%	
Secondary education level	6	18.8%	
Bachelor or higher degree	19	59.4%	
**Sjögren’s disease characteristics**			
Disease organ involvement (n, %)			
Skin	11	34.4%	32
Rheumatological	13	40.6%	32
Neurological	5	15.6%	32
Lung	7	21.9%	32
Cardiac	2	6.3%	32
ANA positivity (n, %)	24	75.0%	32
Anti-SSa and/or anti-SSb positivity (n, %)	12	37.5%	32
Salivary gland biopsy positivity (n, %)	26	86.6%	30
Past or current immunosuppressive treatments (n, %)			
Hydroxychloroquine	14	43.7%	32
Methotrexate	1	3.1%	32
Salazopyrine	1	3.1%	32
**Disease activity scores**			
ESSPRI (mean ± SD, median [IQR])	6.2 ± 2.0	7 [2.7–9]	27
ESSDAI (mean ± SD, median [IQR])	2.5 ± 3.8	0 [0–13]	32
**Cardiovascular risk factors**			
Tobacco use (n, %)	2	6.3%	32
High blood pressure (n, %)	8	25.0%	32
Diabetes (n, %)	3	9.4%	32
Stroke (n, %)	0	0.0%	32

*n* number of patients, *ANA* antinuclear Antibody, *ESSDAI* EULAR Sjögren's syndrome disease activity index, *ESSPRI* EULAR Sjogren's syndrome patient reported index, *IQR* interquartile range.

### Neuropsychological assessments

**Figure 1:**
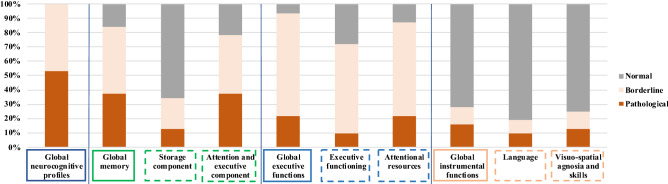
Distribution of neurocognitive profiles of 32 primary Sjögren’s syndrome patients with memory complaints.

Eighty-six percent of patients presented an abnormal memory complaint according to Mac Nair scale or Cognitive complaint questionnaire. A cognitive impairment was identified in all patients (Table 2). In total, 84% of patients exhibited borderline or pathological scores for memory functions, 94% for executive functions, and 30% for instrumental functions. Fifty-three percent of patients exhibited a pathological neurocognitive profile in at least one cognitive function. Thirty-seven percent of them exhibited pathological memory functions, 22% pathological executive functions, and 17% pathological instrumental functions (some exhibited overlapping profiles) (Fig. 1). The praxis were not significantly affected, apart from the visuoconstructive praxis for a few subjects, probably due to impaired visuospatial skills. Details including thresholds values of each test are shown in Supplementary Table 2.

**Table 2:** Neurocognitive profiles of primary Sjögren’s syndrome patients with memory complaints: global cognitive abilities, memory functions abilities, executive functions abilities, instrumental functions abilities.

Cognitive assessments	Number of studied patients	Patients with borderline scores (n, %)	Patients with pathological scores (n, %)	Patients with borderline or pathological scores (n, %)
Global neurocognitive profiles impairment (integrating memory, executive and instrumental functions)	32	15 (47%)	17 (53%)	32 (100%)
**Memory functions impairment**				
Global memory functions	32	15 (47%)	12 (37%)	27 (84%)
Storage component	32	7 (22%)	4 (12%)	11 (34%)
Attention and executive component	32	13 (41%)	12 (37%)	25 (78%)
**Executive functions impairment**				
Global executive functions	32	23 (72%)	7 (22%)	30 (94%)
Executive functioning	32	20 (63%)	3 (9%)	23 (72%)
Attentional resources	32	21 (66%)	7 (22%)	28 (88%)
**Instrumental functions impairment**				
Global instrumental functions	30	4 (13%)	5 (17%)	9 (30%)
Language	30	3 (10%)	3 (10%)	6 (20%)
Visuospatial agnosia and skills	30	4 (13%)	4 (13%)	8 (26%)

*n* number of patients. Global memory functions were assessed through: (1) storage component (part of Free and Cued Reminding Selective Test, FCRST) and (2) attention and executive components (combining FCRST and digit span results). Executive functions evaluation was performed via Digit Symbol Substitution Test (subpart of Wechsler Adult Intelligence Scale-Fourth Edition, WAIS-IV), Trail making test (TMT), Rey-Osterrieth complex figure test, Verbal Fluency Test (VFT), and Stroop Color and Word and Interference Test. Instrumental functions were tested through praxis’ test, Visual Object and Space Perception (VOSP), and picture naming test (D080).

Global memory impairment was confirmed when at least one of its subcomponents was abnormal; global executive functions impairment was confirmed when at least three executive components were abnormal; global instrumental functions impairment was confirmed when at least one of the instrumental components was abnormal.

### Fatigue, pain, psychiatric comorbidities, sleep disorders, and quality of life

**Figure 2:**
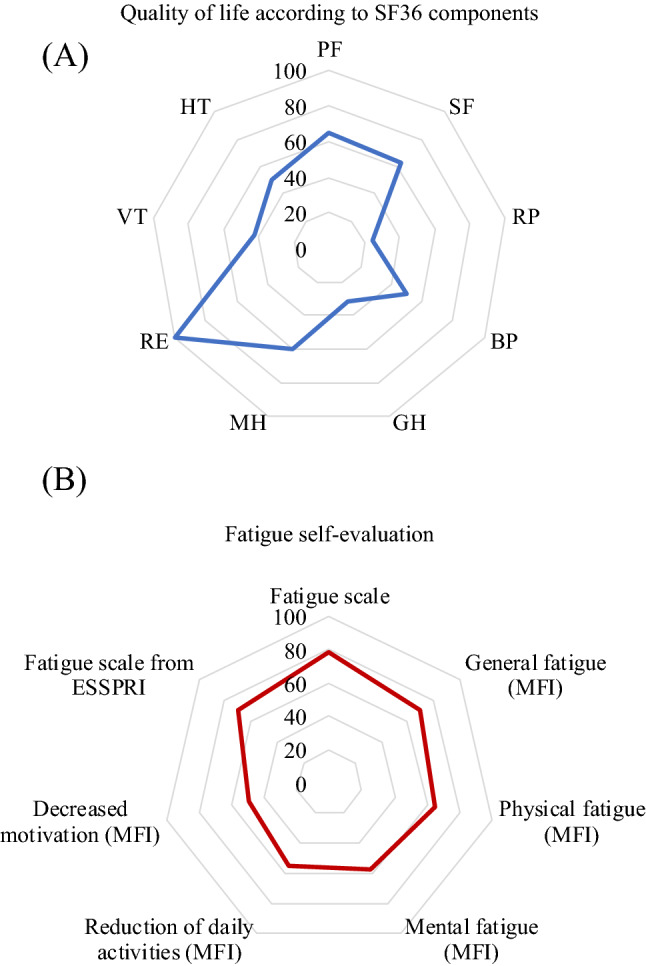
Radar charts of (**A**) patient-reported health-related quality of life and (**B**) fatigue. Short-form 36 evaluation of quality of life, median score of subcomponent (low scores are associated with poor quality of life): *PF* Physical functioning, *SF* social functioning, *RP* role physical (limitations in daily activities associated to physical status), *BP* Bodily pain, *GH* general health, *MH* mental health, *RE* role emotional (limitations in daily activities associated to psychological status), *VT* vitality, *HT* health thinking. Fatigue evaluation according to patients’ self-reported fatigue (median, reported as percentage of total score; high score is associated with higher fatigue); *ESSPRI* EULAR Sjögren’s syndrome patient reported index, *MFI* Multidimensional fatigue inventory and its components. To improve clarity, scores were adjusted to 100.

**Figure 3:**
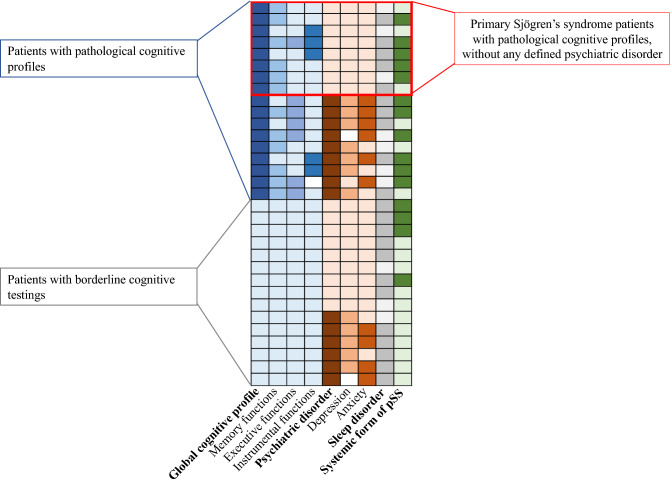
Heatmap showing the distribution of cognitive profiles and associated psychiatric and sleep disorders among 32 primary Sjögren’s patients with cognitive complaints. Darker colours represent worse outcomes. The cluster of 8 patients with cognitive complaints, pathological cognitive disorders, but without anxiety or depression (outlined in red), suggests another underlying pathophysiological mechanism for cognitive troubles. Systemic involvement of the disease is more frequent among patients with pathological cognitive profile.

An abnormal level of fatigue was observed in 32% of pSS, according to the Chalder Fatigue scale (Table 3; Fig. 2B). The median ESSPRI score for pain level was 6/10 [3.5–9]. According to BDI, twelve patients were depressed, 42% with a severe form of depression (Table 3). Forty-eight percent had a mild-to-very severe anxiety trait (≥ 46). In addition, 53% had poor self-esteem. A subgroup of patients (25%) with characterized pathological cognitive profile did not show any psychiatric disorder (Fig. 3).

**Table 3:** Fatigue, psychiatric comorbidities, sleep disorders, and quality of life among a population of primary Sjögren’s syndrome patients with cognitive complaints.

Studied Outcomes			Available data
**Fatigue**			
Abnormal level of fatigue (Chalder Fatigue scale) (n, %)	10	32%	31
MFI (total score) (median, [IQR])	56.5	[38–64]	30
ESSPRI fatigue subscale (median, [IQR])	7	[1–9]	27
**Psychiatric comorbidities**			
Depression (BDI)			
Depression (BDI) (n, %)	12	40%	30
Severe depression (BDI) (n, %)	5	17%	30
Anhedonia (Chapman's scale)			
Physical anhedonia (n, %)	27	93%	29
Social anhedonia (n, %)	8	28%	29
Anxiety trait level (STAI)			
Very weak-to-weak (≤ 45 points) (n, %)	15	52%	29
Mild-to-severe (> 45 points) (n, %)	14	48%	29
**Sleep disorders**			
Excessive daytime sleepiness (Epworth sleepiness scale, n, %)	17	55%	31
Risk of sleep apnea (Berlin questionnaire) (n, %)	14	45%	31
Insomnia (Insomnia Severity Index) (n, %)	22	71%	31
**Quality of life**			
EQ-5D			
VAS (median, [IQR])	60	[50–70]	30
Global score# (median, [IQR])	68.5	[28–80]	30
Poor HR-QoL (n, %)	11	37%	30
HR-QoL worse than death (n, %)	3	10%	30
SF-36			
PCS (mean ± SD)	36.6	± 9.9	32
Pathological PCS (n, %)	16	50%	32
MCS (mean ± SD)	44.1	± 10.8	32
Pathological MCS (n, %)	25	78%	32

*MFI* multidimensional fatigue inventory (a higher score is associated with a high level of fatigue), *ESSPRI* EULAR Sjögren’s syndrome patient reported index, *BDI* beck depression inventory (depression when > 13; severe depression when > 29), Anhedonia (Chapman’s physical anhedonia scale > 24/61; Chapman’s social anhedonia scale > 22/40), *STAI* state trait anxiety inventory, *EQ-5D* EuroQol 5 dimensions of quality of life (< 0.5, mild Qol; < 0, QoL worse than death), *VAS* visual analog scale (EQ-5D VAS from 0 to 100); *HR-Qol* health-related quality of life, *SF-36* short-form 36 evaluation of quality of life (low score for a low level of QoL), *MCS* mental component score, *PCS* physical component score, *IQR* interquartile range; % were adjusted including the number of available data.

Sleep disorders were frequent: 55% had an excessive daytime sleepiness, 45% were considered at risk for sleep apnea, and 71% suffered from insomnia (7 had moderate, and 3 had severe insomnia) (Table 3).

Quality of life was impaired in many patients. According to EQ-5D evaluation, 11 patients described their HR-QoL as mild (0–0.5, n = 8) or worse than death (< 0, n = 3) (Table 3). The Physical Component Score (SF-36 PCS) was worse than the Mental Component Score (SF-36 MCS) (Student test, *p* = 0.007). Most patients complained of severe limitations in daily activities because of physical status, while the objective measures of the disease’s activity (ESSDAI) were not high. They did not feel limited in daily activities by psychological status (Fig. 2A).

### Subgroup analyses: characteristics of the pSS patients with objective global cognitive dysfunction, with poor HR-QoL, or high level of fatigue

We compared the demographical, clinical, neuro-cognitive, and biological evaluations of the 17 pSS patients with pathological global cognitive profiles to the 15 other pSS patients. A systemic involvement of the disease (other than sicca syndrome, skin or joint symptoms) was more frequent in these patients with pathological global cognition (65% vs 27%, *p* = 0.03). However, they did not exhibit more frequent depression, anxiety, sleep disorders, fatigue, pain, or worse QoL (Table 4).

**Table 4:** Clinical, biological and neuropsychological characteristics of patients with pathological cognitive profiles compared to the others.

	pSS patients with pathological global cognitive profile (n = 17)		pSS patients without pathological global cognitive profile (n = 15)		*p* value
**Sjögren's syndrome characteristics**					
Age, years (mean ± SD, median, IQR)	62.2 ± 10.9	53 [44–67]	55.1 ± 12.0	44 [53–70]	0.09^*#*^
SGB positivity (n, %)	13	87%	13	87%	1.00*
Aabs positivity (n, %)	6	35%	6	40%	0.78†
ESSDAI ≥ 4 (n, %)	5	29%	3	20%	0.69*
ESSPRI > 4 (n, %)	10	71%	9	69%	1.00*
Systemic involvement of the disease	11	65%	4	27%	**0.03**†
**Quality of life**					
QoL mild or worse than death (EQ-5D) (n, %)	5	31%	6	43%	0.51†
SF-36 PCS abnormal	9	53%	7	47%	0.72†
SF-36 MCS abnormal	4	24%	3	20%	1.00*
**Fatigue**					
Excessive fatigue (Chalder Fatigue scale) (n, %)	4	24%	6	43%	0.25†
MFI score (mean ± SD, median [IQR])	53.7 ± 19.1	57 [37.5–64]	55.9 ± 17.0	56.5 [53–70]	0.75^*#*^
**Pain**					
ESSPRI (pain subcategory) (mean ± SD, median [IQR])	5.8 ± 3.4	6 [3–9]	6.3 ± 3.0	8 [4–9]	0.80^°^
**Psychiatric disorders**					
Depression (BDI) (n, %)	7	44%	5	36%	0.60†
Mild-to-severe anxiety (STAI trait) (n, %)	6	35%	4	27%	0.60†
**Sleep disorders**					
Excessive daytime sleepiness (Epworth) (n, %)	7	41%	10	71%	0.09†
Risk for sleep apnea (Berlin questionnaire) (n, %)	8	47%	6	43%	0.82†
Insomnia (ISI > 7) (n, %)	12	71%	10	67%	0.99*

*pSS* primary Sjögren’s syndrome, *SGB* salivary gland biopsy, Aabs, autoantibodies (SSa or SSb); *ESSDAI* EULAR Sjögren’s Syndrome disease activity index, *ESSPRI* EULAR Sjogren’s syndrome patient reported index, *Qol* quality of life, *EQ-5D* EuroQol 5 dimensions of quality of life, *SF-36* short-form 36 evaluation of quality of life, *MCS* mental component score, *PCS* physical component score, *BDI* Beck depression inventory, *STAI* state trait anxiety inventory; % were adjusted including the number of available data. Statistical tests: #Student, °Mann–Whitney U-test, †Chi2, *Fisher.

The pSS patients with a poor level of HR-QoL according to EQ-5D had a higher pain level (ESSPRI pain subcategory, *p* < 0.001), a moderate-to-high self-evaluation of the disease activity (ESSPRI score ≥ 4, *p* = 0.01), and a poor physical component score (SF-36, *p* < 0.01). ESSPRI score was inversely correlated to SF-36 PCS (Pearson correlation coefficient − 0.486, *p* = 0.01). However, they did not show higher anxiety level, or more frequent depression, sleep disorder, or excessive fatigue in comparison with patients without poor HR-QoL (Supplementary Table 3).

The patients with higher levels of fatigue were younger (*p* < 0.001), had less systemic involvement of the disease (*p* = 0.03) and fewer dysfunctions in global memory (*p* = 0.046) compared to those with lower level of fatigue (Supplementary Table 4). They showed an increased pain level according to ESSPRI pain subcategory (*p* = 0.02). Their self-evaluation of quality of life (SF-36 MCS) was worse than the self-evaluation of patients with less fatigue. Interestingly, these patients had more excessive daytime sleepiness (*p* = 0.01), and a higher insomnia score (*p* = 0.03) (Supplementary Table 4).

ESSDAI level was not correlated to the observed cognitive disorders, sleep disorders, fatigue or QoL level.

Patients with pathological cognitive profiles did not show more pathological brain MRIs considering Fazekas scores and Scheltens’ scores in comparison with pSS patients without pathological cognitive profiles (*p* = 1 and *p* = 0.36, respectively).

## Discussion

In this study, we analyzed a cohort of pSS patients with cognitive complaints through an extensive neuro-cognitive battery and comorbidities assessments, to decipher the role of each component in the cognitive profile. In accordance with literature data^36^, pSS patients presented mainly memory complaints and more than half of them exhibited pathological scores for global memory, executive functions, or instrumental functions. The proportions of patients with impaired executive functions and visuospatial skills are consistent with previous studies^4,9,10,36^. However, our approach, including a wide range of tests and a personalized comprehensive evaluation of each patient, was more sensitive than previous studies, and allowed an early detection of cognitive impairments (borderline scores), and their interpretation with other cofounding factors. We observed memory impairments in 37% of the patients, which may contribute to an increased risk of developing mild cognitive impairment (MCI), and possibly to the “brain fog” complaints sometimes antedating the diagnosis of pSS^2^. This memory impairment is also consistent with literature data reporting an increased risk for dementia in pSS patients^5,37–40^, especially among pSS patients under age 60 without comorbidities (Taiwanese population-based epidemiological study^41^). However, we did not observe any characterized dementia. These findings illustrate the central position of cognitive impairment in pSS^42,43^, and highlight the significance of patients’ complaints for cognitive frailty. As cognitive frailty is measurable with neuropsychological tests, we consider that cognitive complaint deserves an in-depth investigation in pSS patients to better characterize the cognitive profiles and prognosis, and propose specific strategies.

Since associated psychiatric and sleep disorders greatly interfere with cognitive complaints^44^, we also investigated these conditions in our pSS patients. We found that 47% of all pSS patients with cognitive complaints exhibited a profile of anxiety and/or depression, potentially contributing to cognitive complaints. Our findings are consistent with the literature, in which depression and anxiety are widely described among pSS patients^45–48^. As they are frequent, depression and anxiety should be systematically screened and managed in pSS patients. However, these psychiatric comorbidities alone cannot explain the observed pathological cognitive profile in our study.

We also assessed whether sleep troubles could contribute to cognitive disorders, in addition to anxiety and depression. Indeed, among the 55% of our pSS patients with cognitive complaints but without anxiety or depression, 71% had a sleep disorder. The main troubles were excessive sleepiness, risk of sleep apnea and insomnia. Nevertheless, we did not demonstrate any significant association between sleep disorders and cognitive profiles. Regardless of cognitive profile, sleep troubles are clearly associated with fatigue, another overarching hallmark of pSS^8,49^, that greatly affects pSS patients’ quality of life^6^. In our study, more than one third of patients presented fatigue, which is consistent with previous reports^50^. More interestingly, the fatigue level was highly correlated with excessive daytime sleepiness and insomnia, suggesting a serious impact of sleep troubles on fatigue in pSS patients^51^. Sleep disorders are increased in pSS patients^47^: reduced sleep efficiency, higher rate of awakenings, and higher rate of hypopnea have been described^52,53^. Interestingly, some mechanisms could contribute to hypopnea in pSS, including an obstructive mechanism (airway collapse during sleep already documented in this disease^12^) and a potential dysregulation of central nervous system (more precisely of breathing centers). In our opinion, there is a need for an early diagnosis of sleep disorders in pSS patients, especially in those with a high level of fatigue. For this purpose, the Berlin questionnaire, Epworth sleepiness scale and Insomnia severity index, (internationally validated screening tools), could be easily used in daily practice to identify candidate patients for polysomnography. A potential immunological hypothesis explaining fatigue was not investigated here, and remains to be proven in pSS. However, some studies underlined an inverse correlation between level of fatigue in pSS and pro-inflammatory cytokines, such as interferon-γ-induced protein-10, tumor necrosis factor-α, lymphotoxin-α and interferon-γ^54,55^. These interesting results should not obscure other hypotheses that could contribute to fatigue in pSS, including pain (as demonstrated here), anxiety, depression, or cognitive troubles^56^.

Apart from sleep troubles, cognitive dysfunctions could result from other mechanisms involving immune-mediated processes^54^. In our study, pSS patients with clinical-assessed cognitive impairments exhibited a more pronounced extra-glandular phenotype (excluding skin and joint involvement) than the others. Consistently, Seelinger et al*.* reported that the level of cognitive impairment was correlated to ESSDAI scores, in their series of 64 pSS patients with neurological involvement^36^. Notably, cognitive dysfunction could partially result from encephalitis or multiple-sclerosis-like disorders, whose risk is increased in pSS^57,58^. Furthermore, the interferons (all types) could underlie cognitive dysfunction and probably merit further investigation^59^, since these cytokines are known to be associated with fatigue or depression in pSS^54,60^. A recent study gives weight to neuronal injury, since higher levels of neurofilament light chain protein were found in cerebrospinal fluid (CSF) of pSS patients with visuospatial processing impairments and motor dysfunctions^61^. Taken together, all these findings suggest that cognitive impairment in pSS should be further investigated using new diagnostic tools such as biomarkers (blood, CSF) and/or innovative radiological approaches. Diffusion tensor imaging and resting state functional MRI, focusing on functional connectivity and microstructural changes, would be particularly interesting in this field^62^. Previous studies with more classical imaging techniques (MRI, (99 m)Tc-ECD brain SPECT) have not been able to document clearly the brain inflammatory patterns in pSS, although they demonstrated some abnormalities^63,64^.

Finally, all these symptoms (fatigue, cognitive complaints and sleep disorders) could have contributed to the altered quality of life that we and others observed in pSS. Thus, in our study, the self-evaluation of QoL was mildly-to-severely reduced in one third of our patients^65^. Furthermore, self-evaluation of disease activity (ESSPRI) was correlated to poor HR-QoL (EQ-5D), as reported previously^66^. Our results are also in line with previous findings on the elements of QoL most altered^67^: the physical health (SF-36) was bad among pSS patients, while the level of limitations due to mental health was not severely self-evaluated.

This illustrates the frequently observed discrepancies between subjective symptoms and objective measures in pSS patients^68^. As previously reported in pSS^66^, no relationship was found between the doctors’ evaluation of the disease activity (ESSDAI) and the self-evaluation of quality of life. Moreover, in our study, self-evaluated fatigue (Chalder Fatigue scale) and disease activity (ESSPRI or ESSDAI) were not linked, in accordance with literature^69^. These discrepancies make a comprehensive approach of neurocognitive patterns in pSS methodologically challenging.

The strength of our study was the various methodological approaches used. Our patients underwent a wide range of tests, performed by neuropsychologists, allowing personalized expertise, with a precise cognitive diagnosis for each patient, integrating educational level. This is a sensitive approach to detect early cognitive impairments. However, we only studied a small- sized sample, and included patients from our own hospital recruitment, which is not representative of the global pSS population (especially regarding SGB results and autoantibody positivity). Moreover, we only included patients complaining of cognitive disorders. Our results may thus not be translatable to the general population of pSS patients. This is a preliminary observational study. As an exploratory study, we performed multiple comparison only for descriptive purpose, not for decision-making^70^. Larger studies are needed to confirm these data, especially epidemiological studies integrating psychotropic drug prescriptions among pSS patients. Our approach is difficult to translate in daily practice, since an experienced team of neuropsychologists and physicians cannot be involved routinely. Thus, we would recommend to perform firstly a screening of pSS patients for depression and anxiety with routine tools such as STAI or BDI. Other already validated tools exploring cognitive complaints (such as Montreal cognitive assessment (MoCa) or Brief Cognitive Symptoms Inventory (BCSI)) could also be used, and probably in an easier way for daily practice^71^.

## Conclusion

Cognitive complaints and fatigue concern a large proportion of pSS patients and represent complex clinical situations. Here, we found that global memory, executive functioning and attentional resources are objectively impaired in pSS patients with cognitive complaints. However, their tight interplay with depression and anxiety should be considered by physicians in daily practice and may require psychological or even psychiatric management. Screening for sleep disorders could be helpful in patients with fatigue or cognitive complaints. Further studies are required to validate our proposals for screening and management and should also include extensive cognitive evaluation, and maybe biomarkers or specific brain imaging (measuring brain functional connectivity and microstructural changes).

## Supplementary Information

Supplementary Information.

## Data Availability

Detailed data are available on reasonable request to the corresponding author.
