# Supplementary material for: The interplay between cognition, depression, anxiety, and sleep in primary Sjogren’s syndrome patients
Source: Sci Rep. 2022 Aug 1;12:13176. doi: 10.1038/s41598-022-17354-1 (PMC9343365; doi:10.1038/s41598-022-17354-1)
Supplement: Supplementary file 1 — Supplementary Information. [file 41598_2022_17354_MOESM1_ESM.docx]

**Supplementary Table 1: Test names, subparts, and references.**

| **Type of evaluation** | **Test** |  | **Reference** |
| --- | --- | --- | --- |
| Socio-cultural level | Poitrenaud scale |  | ^1^ |
| **Disease activity** |  |  |  |
| Physician’s evaluation | EULAR Sjögren’s Syndrome Disease Activity Index (ESSDAI) |  | ^2^ |
| Patient’s evaluation | EULAR Sjögren’s Syndrome Patient Reported Index (ESSPRI) |  | ^2^ |
| **Cognitive tests** |  |  |  |
| Cognitive complaints | French cognitive complaint questionnaires (QPC) |  | ^3^ |
| Global cognitive dysfunction screening | Mini-mental state (MMSE) |  | ^4^ |
| Memory functions testing | Free and Cued Reminding Selective Test (FCRST) |  | ^5^ |
| Executive functioning of the memory | Digit span |  |  |
| Executive function tests | Digit Symbol Substitution Test (DSST), subpart of Wechsler Adult Intelligence Scale-Fourth Edition, WAIS-IV) |  | ^6^ |
|  | Trail making test (TMT) |  | ^7^ |
|  | Rey-Osterrieth complex figure test |  | ^8^ |
|  | Verbal Fluency Test (VFT) |  |  |
|  | Categorical Naming Test (CNT) |  | ^9^ |
|  | Stroop Color and Word and Interference Test |  | ^10^ |
| Instrumental functions testing | Visual Object and Space Perception (VOSP) |  | ^11^ |
|  | Picture-naming test (DO-80) |  | ^12^ |
| Praxis’ test |  |  | ^13^ |
| **Fatigue** |  |  |  |
|  | Chalder Fatigue scale |  | ^14^ |
|  | Multidimensional Fatigue Inventory (MFI) |  | ^15^ |
|  | ESSPRI fatigue subscale |  | ^2^ |
| **Pain** | ESSPRI pain subscale |  | ^2^ |
| **Psychiatric comorbidities** |  | |  |
| Depression (BDI) | Beck Depression Inventory version II (BDI) | | ^16^ |
| Anhedonia | Chapman’s scale | | ^17^ |
| Anxiety | State Trait Anxiety Inventory (STAI) | | ^18^ |
| Self-esteem | Rosenberg self-esteem scale | | ^19,20^ |
| **Sleep disorders** | | |  |
| Excessive daytime sleepiness | Epworth sleepiness scale (ESS) | | ^21^ |
| Risk of sleep apnoea | Berlin questionnaire | | ^22^ |
| Insomnia | Insomnia Severity Index (ISI) | | ^23^ |
| **Quality of life** |  |  |  |
| Health-related quality of life (HR-QoL) | EuroQol 5 Dimensions (EQ-5D) | | ^24,25^ |
| Quality of life | Short-Form 36 (SF-36) | | ^26^ |
| **Brain MRI** |  | |  |
| Brain atrophy and white matter hyperintensity lesions | Fazekas’ scale and Scheltens’ score | | ^27^ |

*EULAR, EUropean League Against Rheumatism.*

References:

1. Poitrenaud, J., Eustache, F. & Agniel, A. Neuropsychologie Clinique des démences : Evaluations et prise en charge. (Marseille : Solal, 1995).

2. Seror, R. et al. Validation of EULAR primary Sjögren’s syndrome disease activity (ESSDAI) and patient indexes (ESSPRI). Ann. Rheum. Dis. 74, 859–866 (2015).

3. Thomas-Anterion, C., HONORE - MASSON, S., Berne, G., Ruel, J. & Laurent, B. Le questionnaire de plainte cognitive (QPC) : un outil de recherche de plainte suspecte d’évoquer une maladie d’Alzheimer. L’année gérontologique 56–65 (2003).

4. Folstein, M. F., Folstein, S. E. & McHugh, P. R. ‘Mini-mental state’. A practical method for grading the cognitive state of patients for the clinician. J Psychiatr Res 12, 189–198 (1975).

5. Van der Linden, M. et al. L’épreuve de rappel libre/rappel indicé à 16 items (RL/RI-16). vol. L’évaluation des troubles de la mémoire. Présentation de quatre tests de mémoire épisodique (avec leur étalonnage) (Marseille : Solal, 2004).

6. Wechsler, D. Wechsler Adults Intelligence Scale WAIS-III. (Paris : Editions du Centre de Psychologie Appliquée, 2000).

7. Tombaugh, T. N. Trail Making Test A and B: normative data stratified by age and education. Arch Clin Neuropsychol 19, 203–214 (2004).

8. Berry, D., Allen, R. & Schmitt, F. Rey-Osterrieth complex figure: Psychometric characteristics in a geriatric sample. Clin Neuropsychol. 5(2):143-53 (1991).

9. Cardebat, D., Doyon, B., Puel, M., Goulet, P. & Joanette, Y. [Formal and semantic lexical evocation in normal subjects. Performance and dynamics of production as a function of sex, age and educational level]. Acta Neurol Belg 90, 207–217 (1990).

10. Stroop, J. Studies of interference in serial verbal reactions. J Exp Psychol. 18(6):643-62 (1935).

11. Warrington, E. K. & Merle, J. The visual object and space perception battery. (Bury St. Edmunds : Thames Valley Test Company., 1991).

12. Deloche, G. & Hannequin, D. Test de dénomination orale d’images. Paris: les Editions du Centre de Psychologie Appliquée.

13. Peigneux, P. et al. [A neuropsychological and functional brain imaging study of visuo-imitative apraxia]. Rev Neurol (Paris) 156, 459–472 (2000).

14. Chalder, T. et al. Development of a fatigue scale. J Psychosom Res 37, 147–153 (1993).

15. Schwarz, R., Krauss, O. & Hinz, A. Fatigue in the general population. Onkologie 26, 140–144 (2003).

16. Beck, A. T., Ward, C. H., Mendelson, M., Mock, J. & Erbaugh, J. An inventory for measuring depression. Arch Gen Psychiatry 4, 561–571 (1961).

17. Kosmadakis, C. S., Bungener, C., Pierson, A., Jouvent, R. & Widlöcher, D. [Translation and validation of the Revised Social Anhedonia Scale (SAS Social Anhedonia Scale, M.L. Eckblad, L.J. Chapman et al., 1982). Study of the internal and concurrent validity in 126 normal subjects]. Encephale 21, 437–443 (1995).

18. Spielberger, C. D. State-Trait Anxiety Inventory for Adults Sampler Set - Manual, Instrument and Scoring Guide. (Mind Garden, Inc, 1983).

19. Rosenberg, M. Society and the adolescent self-image. (Princeton University Press, 1965).

20. Gray-Little, B., Williams, V. & Hancock, T. An itemresponse theory analysis of the Rosenberg Self-Esteem Scale. 23, 443–451 (1997).

21. Johns, M. W. A new method for measuring daytime sleepiness: the Epworth sleepiness scale. Sleep 14, 540–545 (1991).

22. Netzer, N. C., Stoohs, R. A., Netzer, C. M., Clark, K. & Strohl, K. P. Using the Berlin Questionnaire to identify patients at risk for the sleep apnea syndrome. Ann Intern Med 131, 485–491 (1999).

23. Bastien, C. H., Vallières, A. & Morin, C. M. Validation of the Insomnia Severity Index as an outcome measure for insomnia research. Sleep Med 2, 297–307 (2001).

24. Hanly, J. G. et al. Neuropsychiatric events in systemic lupus erythematosus: a longitudinal analysis of outcomes in an international inception cohort using a multistate model approach. Ann Rheum Dis 79, 356–362 (2020).

25. Brooks, R. G., Jendteg, S., Lindgren, B., Persson, U. & Björk, S. EuroQol: health-related quality of life measurement. Results of the Swedish questionnaire exercise. Health Policy 18, 37–48 (1991).

26. Louthrenoo, W., Kasitanon, N., Morand, E. & Kandane-Rathnayake, R. Comparison of performance of specific (SLEQOL) and generic (SF36) health-related quality of life questionnaires and their associations with disease status of systemic lupus erythematosus: a longitudinal study. Arthritis Res Ther 22, 8 (2020).

27. Scheltens, P. et al. White matter changes on CT and MRI: an overview of visual rating scales. European Task Force on Age-Related White Matter Changes. Eur Neurol 39, 80–89 (1998).

**Supplementary Table 2: thresholds and details of the results of neurocognitive subtests** for global cognitive abilities, memory abilities, instrumental abilities and executive functions abilities among a population of pSS with memory complaints.

|  | Tests | Subtests | N | Median | [Min-Max] | IQR | Mean (+/- SD) | Patients with borderline results (n, %) | Patients with pathological results (n, %) | Test thresholds |
| --- | --- | --- | --- | --- | --- | --- | --- | --- | --- | --- |
| Global cognitive dysfunction screening | MMSE |  | 32 | 28 | 21-30 | 26-29 | 27.2 ± 2.6 | 9 (28.1%) | 8 (25.0%) | Borderline < 10th percentile / Pathological < 5th percentile |
| Memory functions testing | FCRST | 3 Learning free recall | 32 | 32 | 22-43 | 28-34 | 31.1 ± 4.8 | 9 (28.1%) | 4 (12.5%) | Borderline: Z score < -1 |
|  |  | 3 Learning total recall | 32 | 47 | 40-48 | 45-48 | 46.2 ± 2.4 | 4 (12.5%) | 1 (3.1%) | Pathological: Z score < -1.65 |
|  |  | Late total recall | 31 | 16 | 14-16 | 16-16 | 15.8 ± 0.6 | 5 (16.1%) | 2 (6.5%) |  |
| Executive functioning of the memory | Digit span | Forward | 32 | 5 | 4-9 | 5-6 | 5.5 ± 1.1 | 18 (56.3%) | 5 (15.6%) | Borderline: 5; pathological: 4 |
|  |  | Backward | 32 | 4 | 3-8 | 4-5 | 4.5 ± 1.2 | 19 (59.4%) | 6 (18.8%) | Borderline: 4; pathological: 3 |
|  | WAIS-IV (Digit Symbol Substitution Test) |  | 31 | 9 | 5-18 | 8-11 | 9.6 ± 3.1 | 6 (19.4%) | 4 (12.9%) | Borderline: note  standard < 8 |
|  |  |  |  |  |  |  |  |  |  | Pathological: note standard < 6 |
|  | TMT | A part | 32 | 42.5 | 22-86 | 34-48 | 43.7 ± 14.5 | 0 (0%) | 2 (6.3%) | Borderline: average < 10th percentile (compared to a reference population) |
|  |  | B part | 31 | 104 | 43-192 | 77-123 | 104.8 ± 35.2 | 7 (21.9%) | 2 (6.3%) | Pathological: average < 5th percentile |
| Executive function tests |  | TMTB-A | 31 |  |  |  |  | 5 (15.6%) | 2 (6.3%) |  |
|  | Rey Complex Figure Test | Z | 31 | 34 | 17.5-36 | 31-35 | 32.4 ± 4.2 | 5 (16.1%) | 3 (9.7%) | Borderline: Z score < -1 |
|  |  | Type | 31 | 1 | 1-5 | 1-2 | 1.71 ± 1.16 | 6 (19.4%) | 1 (3.2%) | Pathological: Z score < -1.65 |
|  | VFT | Letter fluency | 31 | 20 | 12-35 | 18-24 | 21.0 ± 5.8 | 6 (19.4%) | 2 (6.5%) | Borderline: Z score < -1 |
|  |  | Category fluency | 32 | 30 | 19-42 | 23-32 | 28.7 ± 5.7 | 7 (21.5%) | 2 (6.3%) | Pathological: Z score < -1.65 |
|  | Stroop | Stroop W | 31 | 91 | 67-118 | 84.5-104 | 93.3 ± 13.6 | 8 (25.8%) | 1 (3.2%) | Number of identified letters < 17 |
|  |  | Stroop C | 31 | 70 | 44-91 | 59.5-78.5 | 68.7 ± 13.8 | 9 (29.0%) | 2 (6.5%) | Number of correct figures-points matching <7 |
|  |  | Stroop CW | 31 | 38 | 17-58 | 30-46.5 | 38.6 ± 11.5 | 9 (29.0%) | 1 (3.2%) |  |
|  |  | Stroop I | 31 | 0.2 | -1.4-2.1 | -0.3-0.73 | 0.18 ± 0.79 | 3 (9.7%) | 0 (0%) |  |
|  | VOSP | Incomplete letter | 31 | 20 | 17-20 | 19-20 | 19.3 ± 0.8 | 1 (3.2%) | 0 (0%) | Number of identified letters < 17 |
| Instrumental functions testing |  | Number Location | 30 | 9 | 6-10 | 8-10 | 8.9 ± 1.2 | 4 (13.3%) | 2 (6.7%) | Number of correct figures-points matching <7 |
|  | Picture-naming test (DO-80) |  | 30 | 79 | 66-80 | 78-80 | 78.2 ± 2.8 | 5 (16.7%) | 3 (10.0%) | Borderline: Z score < -1; Pathological: Z score < -1.65 |

*IQR, interquartile range; MMSE, Mini mental state evaluation; FCRST, French version of the Free and Cued Reminding Selective Test; TMT, Trail making test; WAIS-IV, Wechsler Adult Intelligence Scale-Fourth Edition; Rey Complex Figure Test; VFT, Verbal Fluency Test; Stroop W, word test; Stroop C, Color test; Stroop CW, color-word test; Stroop I, Interference; VOSP, Visual Object and Space Perception test battery; DO80, oral denomination 80.*

**Supplementary Figure 1: Flow-chart of the selection of primary Sjögren’s syndrome patients with cognitive complaints involved in the study**

**Supplementary Table 3: Clinical, biological and neuropsychological characteristics of primary Sjögren’s syndrome patients according to their level of self-reported health-related quality of life.**

|  | pSS patients with poor EQ-5D score (n=11) | | pSS patients without poor EQ-5D score (n=19) | |  |  |
| --- | --- | --- | --- | --- | --- | --- |
|  |  |  |  |  | p value |  |
| **Sjögren's syndrome characteristics** | | |  |  |  |  |
| Age, years (mean±SD, median, [IQR]) | 57.9±14.5 | 61 [44-70] | 60.1±10.8 | 59.0 [52-69] | 0.64*^#^* |  |
| SGB positivity (n, %) | 9 | 81.8% | 16 | 88.9% | 0.62* |  |
| Aabs positivity (n, %) | 5 | 45.5% | 6 | 31.6% | 0.70† |  |
| ESSDAI ≥ 4 (n, %) | 4 | 36.4% | 3 | 15.8% | 0.37* |  |
| ESSPRI > 4 (n, %) | 10 | 100.0% | 9 | 52.9% | **0.01*** |  |
| Systemic involvement of the disease | 4 | 36.4% | 10 | 52.6% | 0.39† |  |
| **Quality of life** |  |  |  |  |  |  |
| SF-36 PCS abnormal | 10 | 90.1% | 6 | 31.6% | **0.002**† |  |
| SF-36 MCS abnormal | 4 | 36.4% | 3 | 15.8% | 0.37* |  |
| **Fatigue** |  |  |  |  |  |  |
| Excessive fatigue (Fatigue scale) (n, %) | 6 | 54.5% | 4 | 21.1% | 0.11† |  |
| MFI score (mean±SD, median [IQR]) | 60.2±22.8 | 59.5 [38-80] | 52.7±14.6 | 55 [45-64] | 0.29*^#^* |  |
| **Pain** |  |  |  |  |  |  |
| ESSPRI (pain subcategory) (mean±SD, median [IQR]) | 8.9±0.74 | 9 [8-9] | 4.3 ±2.72 | 4 [2.5-6] | **<0.001°** |  |
| **Psychiatric disorders** | | |  |  |  |  |
| Depression (BDI) (n, %) | 7 | 63.7% | 5 | 26.3% | 0.06† |  |
| Mild-to-severe anxiety (STAI trait) (n, %) | 6 | 54.6% | 8 | 42.1% | 0.70† |  |
| **Sleep disorders** | | |  |  |  |  |
| Excessive daytime sleepiness (Epworth) (n, %) | 7 | 63.7% | 10 | 52.6% | 0.56† |  |
| Risk for sleep apnoea (Berlin questionnaire) (n, %) | 7 | 63.7% | 7 | 36.8% | 0.16† |  |
| Insomnia (ISI >7) (n, %) | 9 | 81.9% | 13 | 68.4% | 0.67* |  |

*Poor level of health-related quality of life was defined with a global score < 0.5 in EQ-5D (EuroQol 5 Dimensions of quality of life). pSS, primary Sjögren's syndrome; SGB, salivary gland biopsy; Aabs, autoantibodies (SSa or SSb); ESSDAI, EULAR Sjögren's Syndrome Disease Activity Index; ESSPRI, EULAR Sjogren's Syndrome Patient Reported Index; Qol, Quality of life; SF-36, short-form 36 evaluation of quality of life; MCS, mental component score; PCS, physical component score; BDI, Beck Depression Inventory; STAI, State Trait Anxiety Inventory; % were adjusted including the number of available data. Statistical tests: #Student, °Mann–Whitney U-test, †Chi2, *Fisher.*

**Supplementary Table 4: Clinical, biological and neuropsychological characteristics of primary Sjögren’s syndrome patients according to their fatigue level (fatigue scale).**

|  | pSS patients with pathological fatigue score (n=10) | | pSS patients without pathological fatigue score (n=21) | |  |
| --- | --- | --- | --- | --- | --- |
|  |  |  |  |  | p value |
| **Sjögren's syndrome characteristics** | | |  |  |  |
| Age, years (mean±SD, median, IQR) | 47.7±7.3 | 46 [42-52] | 64.6±9.6 | 67.0 [57-70] | **<0.001***^#^* |
| SGB positivity (n, %) | 7 | 70.0% | 18 | 94.7% | 0.10*** |
| Aabs positivity (n, %) | 5 | 50.0% | 7 | 33.3% | 0.45*** |
| ESSDAI ≥ 4 (n, %) | 1 | 10.0% | 7 | 33.3% | 0.22*** |
| ESSPRI > 4 (n, %) | 9 | 90.0% | 10 | 58.8% | 0.19*** |
| Systemic involvement of the disease | 2 | 20.0% | 13 | 61.9% | **0.03**† |
| **Neurocognitive dysfunction** | | |  |  |  |
| Global memory functions | 1 | 10.0% | 11 | 52.4% | **0.046***** |
| Global executive functions | 2 | 20.0% | 5 | 23.8% | 1*** |
| Global instrumental functions | 2 | 20.0% | 3 | 14.3% | 1*** |
| **Quality of life** |  |  |  |  |  |
| QoL mild or worse than death (EQ-5D) (n, %) | 6 | 60.0% | 5 | 25.0% | 0.11*** |
| SF-36 PCS abnormal | 5 | 50.0% | 11 | 52.4% | 0.90† |
| SF-36 MCS abnormal | 5 | 50.0% | 2 | 9.5% | **0.02***** |
| **Fatigue** |  |  |  |  |  |
| MFI score (mean±SD, median [IQR]) | 63.0±17.3 | 63 [52-79] | 50.6±17.0 | 50.5 [38-64] | 0.07*^#^* |
| **Pain** |  |  |  |  |  |
| ESSPRI (pain subcategory) (mean±SD, median [IQR]) | 7.9±2.15 | 9 [8-9] | 5.1 ±3.21 | 5 [3-8] | **0.02°** |
| **Psychiatric disorders** | | |  |  |  |
| Mild-to-severe anxiety (STAI trait) (n, %) | 5 | 50.0% | 9 | 42.9% | 1.00 |
| Depression (BDI) (n, %) | 6 | 60.0% | 6 | 28.6% | 0.12 |
| **Sleep disorders** | | |  |  |  |
| Excessive daytime sleepiness (Epworth) (n, %) | 9 | 90.0% | 8 | 38.2% | **0.01**† |
| Risk for sleep apnea (Berlin questionnaire) (n, %) | 6 | 60.0% | 8 | 38.1% | 0.25† |
| Insomnia (ISI >7) (n, %) | 10 | 100.0% | 12 | 57.1% | **0.03***** |

*Pathological fatigue was determined according to the fatigue scale score, adjusted with age and sex ranges (at least ≥9).* *pSS, Sjögren’s syndrome; SGB, salivary gland biopsy; Aabs, autoantibodies (SSa or SSb); ESSDAI, EULAR Sjögren’s Syndrome Disease Activity Index; ESSPRI, EULAR Sjogren’s Syndrome Patient Reported Index; Qol, Quality of life; EQ-5D, EuroQol 5 Dimensions of quality of life; SF-36, short-form 36 evaluation of quality of life; MCS, mental component score; PCS, physical component score; BDI, Beck Depression Inventory; STAI, State Trait Anxiety Inventory; % were adjusted including the number of available data. Statistical tests: #Student, °Mann–Whitney U-test, †Chi2, *Fisher.*
